# 3-Nitro­benzoic acid–3-methyl-4-nitro­pyridine 1-oxide (1/1)

**DOI:** 10.1107/S1600536809023563

**Published:** 2009-06-24

**Authors:** Rodolfo Moreno-Fuquen, Javier Ellena, Jahyr E. Theodoro

**Affiliations:** a Departamento de Química - Facultad de Ciencias, Universidad del Valle, Apartado 25360, Santiago de Cali, Colombia; b Instituto de Física, IFSC, Universidade de São Paulo, São Carlos, Brazil

## Abstract

The title adduct, C_7_H_5_NO_4_·C_6_H_6_N_2_O_3_, forms part of an ongoing study of the design of non-centrosymmetric systems based on 3-methy-4-nitro­pyridine 1-oxide. The components of the adduct are linked by inter­molecular O—H⋯O hydrogen bonds. The rings of the two components are nearly planar, with a dihedral angle of 11.9 (2)° between the planes. The supra­molecular structure shows that mol­ecules of the title complex are linked into sheets by a combination of strong O—H⋯O and weak C—H⋯O hydrogen bonds.

## Related literature

For background information on the non-linear optical properties of 3-methyl-4-nitro­pyridine 1-oxide (POM) see: Sapriel *et al.* (1989 ▶). For the free mol­ecular systems POM and 3-nitro­benzoic acid (NBA), see: Baert *et al.* (1988 ▶); Dhaneshwar *et al.* (1975 ▶). For hydrogen bonding, see: Etter (1990 ▶); Emsley (1984 ▶). For a related structure, see: Moreno-Fuquen *et al.* (2002 ▶).
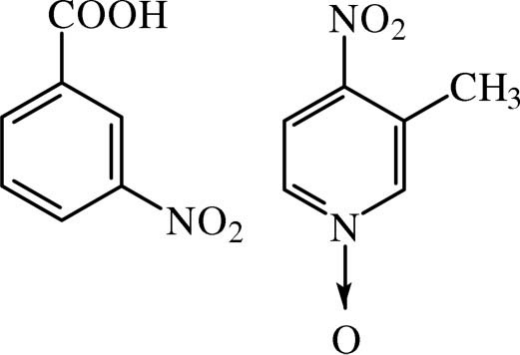


## Experimental

### 

#### Crystal data

C_7_H_5_NO_4_·C_6_H_6_N_2_O_3_
*M* *_r_* = 321.25
Monoclinic, 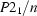

*a* = 7.1221 (4) Å
*b* = 11.0660 (2) Å
*c* = 17.9921 (4) Å
β = 98.170 (4)°
*V* = 1403.62 (9) Å^3^
*Z* = 4
Mo *K*α radiation
μ = 0.13 mm^−1^
*T* = 291 K
0.20 × 0.18 × 0.15 mm

#### Data collection

Enraf–Nonius CAD-4 diffractometer
Absorption correction: ψ scan (North *et al.*, 1968 ▶) *T*_min_ = 0.961, *T*_max_ = 0.985
3668 measured reflections
2481 independent reflections
1511 reflections with *I* > 2σ(*I*)
*R*_int_ = 0.072
2 standard reflections frequency: 120 min intensity decay: 1.1%

#### Refinement

*R*[*F* ^2^ > 2σ(*F* ^2^)] = 0.052
*wR*(*F* ^2^) = 0.191
*S* = 1.04
2481 reflections
214 parameters
H atoms treated by a mixture of independent and constrained refinement
Δρ_max_ = 0.38 e Å^−3^
Δρ_min_ = −0.26 e Å^−3^

### 

Data collection: *CAD-4 Software* (Enraf–Nonius, 1989 ▶); cell refinement: *CAD-4 Software*; data reduction: *XCAD4* (Harms & Wocadlo, 1995 ▶); program(s) used to solve structure: *SHELXS97* (Sheldrick, 2008 ▶); program(s) used to refine structure: *SHELXL97* (Sheldrick, 2008 ▶); molecular graphics: *ORTEP-3 for Windows* (Farrugia, 1997 ▶); software used to prepare material for publication: *PARST95* (Nardelli, 1995 ▶).

## Supplementary Material

Crystal structure: contains datablocks I, global. DOI: 10.1107/S1600536809023563/bq2143sup1.cif

Structure factors: contains datablocks I. DOI: 10.1107/S1600536809023563/bq2143Isup2.hkl

Additional supplementary materials:  crystallographic information; 3D view; checkCIF report

## Figures and Tables

**Table 1:** Hydrogen-bond geometry (Å, °)

*D*—H⋯*A*	*D*—H	H⋯*A*	*D*⋯*A*	*D*—H⋯*A*
O3—H31⋯O5^i^	0.86 (5)	1.73 (6)	2.578 (3)	170 (5)
C10—H10⋯O4^ii^	0.93	2.48	3.351 (4)	156
C2—H2⋯O1^iii^	0.93	2.55	3.371 (4)	148
C9—H9⋯O2^iv^	0.93	2.39	3.260 (4)	156
C13—H131⋯O3^v^	0.96	2.67	3.502 (4)	146
C11—H11⋯O5^vi^	0.93	2.40	3.270 (4)	156
C13—H132⋯O7^vii^	0.96	2.62	3.475 (4)	148

Symmetry codes: (i) 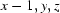; (ii) 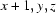; (iii) 
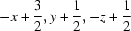
; (iv) 
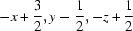
; (v) 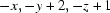; (vi) 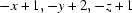; (vii) 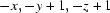.

**Fig. 1.**
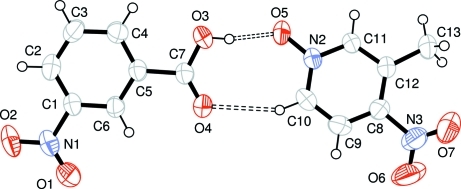
An ORTEP-3 (Farrugia, 1997) plot of the title compound with the atomic labeling scheme. Displacement ellipsoids are drawn at the 50% probability level. Dashed lines show H-bonds.

**Fig. 2.**
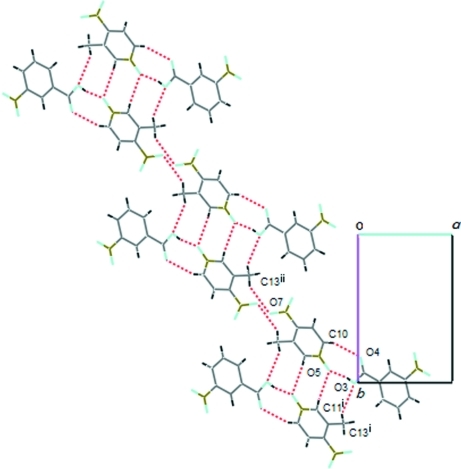
Part of the crystal structure of (I), showing the formation of R22(8), R23(8) and R22(12) centrosymmetric rings along [001]. Symmetry codes: (i) -1 - x, 2 - y, 1 - z; (ii) -2 - x, 1 - y, 1 - z.

**Fig. 3.**
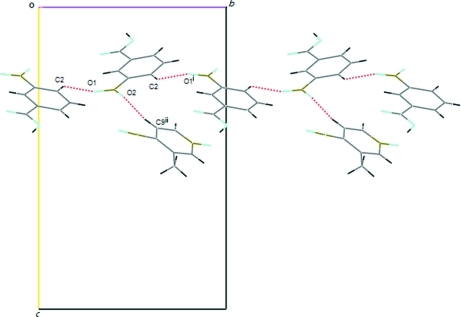
Part of the crystal structure of (I), showing the formation of C(5) chains along [100]. Symmetry codes: (i) -x + 3/2, y + 1/2, 1/2 - z; (ii) -x + 1/2, y - 1/2, -z + 1/2.

## supplementary crystallographic information

### Comment

Within the family of crystal compounds having the N-oxide group, the 3-methyl-4-nitropyridine 1-oxide (POM) is one of the best electro-optic materials in the visible range (Sapriel *et al.*, 1989). The title work is part of a series of structural studies on molecular complexes, formed by hydrogen bonds, with potential non-linear optical applications (Moreno-Fuquen *et al.*, 2002). To complement the crystallographic information available on compounds based on POM complexes, to study its supramolecular behaviour, and to analyze the type of hydrogen-bonds formed in the adduct, a determination of the 3-nitrobenzoic acid (NBA) and POM, adduct (I), was undertaken. The free molecular systems POM and NBA have been reported in the literature (Baert *et al.*, 1988; Dhaneshwar *et al.*, 1975), respectivelly, and they can be used as reference systems to compare with the structural characteristics of (I). The title complex (I) is held together by a strong intermolecular hydrogen bond (Emsley, 1984) between the N—O group of the POM molecule and the O—H group of the NBA molecule (Table 1.). A displacement ellipsoid plot of (I), with the atomic numbering scheme is shown in Figure 1. The title compound shows an O3···O5 bond length of 2.578 (3) Å and O3—H31···O5 bond angle of 170 (5)°. The dihedral angle between the planes of the POM and MABA rings of (I) is 11.9 (2)°. The presence of an additional C10—H10···O4 intermolecular hydrogen bond allows the formation of *R*_2_^2^(8) rings between the molecules in the adduct (I). The free NBA and other organic acids usually exist as dimeric pairs forming *R*_2_^2^(8) rings between the molecules. In a first stage, for the formation of the adduct (I), the NBA molecule breaks the hydrogen bond compromised in the formation of the dimer and subsequently the NBA molecule fits the C—O bonds in order to form the new *R*_2_^2^(8) ring with the POM molecule. Indeed, from symmetric C—O bonds (C···O = 1.256 (4) Å) in the free NBA molecule (Dhaneshwar *et al.*, 1975), the C—O bond lengths change to 1.205 (3) Å and 1.311 (4) Å in the adduct (I). Additionally, the C(5)—C(7) bond length in the NBA free molecule changes from 1.488 (3) Å to 1.502 (4) Å, as a result of the formation of the title complex. The molecules which form the title adduct (I), are linked into sheets by a combination of O—H···O and C—H···O hydrogen bonds (Table 1) and these interactions define the bulk structure of the crystal. In a first substructure, the formation of a centrosymmetric rings generated by pairs of O—H···O and C—H···O hydrogen bonds is observed (Fig. 2). The carboxyl O3 atom of NBA molecule acts as hydrogen bond donor to N-oxide atom O5 of POM, in the molecule at (*x*, *y*, *z*) and C10 atom of POM in the molecule at (*x*, *y*, *z*) acts as hydrogen bond donor to carboxyl atom O4 in the molecule at (*x*,*y*,*z*) so forming by inversion a centrosymmetric *R*_2_^2^(8) ring (Etter, 1990). In addition, atoms O3 and O5 in the molecule at (*x*,*y*,*z*) act as hydrogen bond acceptors from the atoms C11 and C13 in the molecule at (-1 - *x*, 2 - *y*, 1 - *z*) respectively, so forming by inversion *R*_2_^3^(8) and *R*_2_^2^(8) edge-fused centrosymmetric rings. Prolonging the growth of the crystal in the [-110] direction, the nitro O7 atom in the molecule at (*x*, *y*, *z*) acts as hydrogen bond acceptor from the atom C13 in the molecule at (-*x* + 1/2, *y* - 1/2,-*z* + 1/2), so forming *R*_2_^2^(12) centrosymmetric ring. In a second substructure, atom C2 in the molecule at (*x*, *y*, *z*) acts as hydrogen bond donor to nitro O1 atom in the molecule at (-*x* + 3/2, 1/2 + *y*, 1/2 - *z*). In addition, atom O2 in the molecule at (*x*, *y*, *z*) acts as hydrogen bond donor to C9 atom in the molecule at (-*x* + 1/2, *y* - 1/2, -*z* + 1/2). The propagation of this interaction forms a C(5) (Etter, 1990) chain running along [010] direction (Fig. 3). The combination of these substructures is sufficient to generate a continuous framework structure. The presence of a centre of symmetry in the crystal inhibits the non-linear optical response.

### Experimental

The synthesis of the title compound (I) was carried out by slow evaporation of equimolar quantities of 3-nitrobenzoic acid (0.805 g, 0.0048 mol) and 3-methyl-4-nitropyridine 1-oxide (0.739 g) in 150 ml of dry acetonitrile. Pale-yellow prisms of good quality, suitable for x-ray analysis with a melting point of 589 (1) K were obtained. The initial reagents were purchased from Aldrich Chemical Co., and were used without additional purification.

### Refinement

All H-atoms were located from difference Fourier maps and then they were treated as riding atoms [C_aro_—H= 0.93 A°, and C_sp3_—H= 0.96 A°, *U*_iso_(H)=1.2*U*_eq_(C_aro_), *U*_iso_(H)= 1.5*U*_eq_(C_sp3_)).

### Crystal data

**Table:**

C_7_H_5_NO_4_·C_6_H_6_N_2_O_3_	*F*(000) = 664
*M**_r_* = 321.25	*D*_x_ = 1.520 Mg m^−^^3^
Monoclinic, *P*2_1_/*n*	Melting point: 589(1) K
Hall symbol: -P 2yn	Mo *K*α radiation, λ = 0.71073 Å
*a* = 7.1221 (4) Å	Cell parameters from 23 reflections
*b* = 11.0660 (2) Å	θ = 4.9–8.7°
*c* = 17.9921 (4) Å	µ = 0.13 mm^−^^1^
β = 98.170 (4)°	*T* = 291 K
*V* = 1403.62 (9) Å^3^	Prism, pale-yellow
*Z* = 4	0.20 × 0.18 × 0.15 mm

### Data collection

**Table:**

Enraf–Nonius CAD-4 diffractometer	1511 reflections with *I* > 2σ(*I*)
Radiation source: fine-focus sealed tube	*R*_int_ = 0.072
graphite	θ_max_ = 25.0°, θ_min_ = 2.2°
ω/2θ scans	*h* = 0→8
Absorption correction: ψ scan (North *et al.*, 1968)	*k* = 0→13
*T*_min_ = 0.961, *T*_max_ = 0.985	*l* = −21→21
3668 measured reflections	2 standard reflections every 120 min
2481 independent reflections	intensity decay: 1.1%

### Refinement

**Table:**

Refinement on *F*^2^	Secondary atom site location: difference Fourier map
Least-squares matrix: full	Hydrogen site location: inferred from neighbouring sites
*R*[*F*^2^ > 2σ(*F*^2^)] = 0.052	H atoms treated by a mixture of independent and constrained refinement
*wR*(*F*^2^) = 0.191	*w* = 1/[σ^2^(*F*_o_^2^) + (0.0995*P*)^2^ + 0.4787*P*] where *P* = (*F*_o_^2^ + 2*F*_c_^2^)/3
*S* = 1.04	(Δ/σ)_max_ < 0.001
2481 reflections	Δρ_max_ = 0.38 e Å^−^^3^
214 parameters	Δρ_min_ = −0.26 e Å^−^^3^
0 restraints	Extinction correction: *SHELXL97* (Sheldrick, 2008), Fc^*^=kFc[1+0.001xFc^2^λ^3^/sin(2θ)]^-1/4^
Primary atom site location: structure-invariant direct methods	Extinction coefficient: 0.017 (4)

### Special details

**Table:**

Geometry. All e.s.d.'s (except the e.s.d. in the dihedral angle between two l.s. planes) are estimated using the full covariance matrix. The cell e.s.d.'s are taken into account individually in the estimation of e.s.d.'s in distances, angles and torsion angles; correlations between e.s.d.'s in cell parameters are only used when they are defined by crystal symmetry. An approximate (isotropic) treatment of cell e.s.d.'s is used for estimating e.s.d.'s involving l.s. planes.
Refinement. Refinement of *F*^2^ against ALL reflections. The weighted *R*-factor *wR* and goodness of fit *S* are based on *F*^2^, conventional *R*-factors *R* are based on *F*, with *F* set to zero for negative *F*^2^. The threshold expression of *F*^2^ > σ(*F*^2^) is used only for calculating *R*-factors(gt) *etc*. and is not relevant to the choice of reflections for refinement. *R*-factors based on *F*^2^ are statistically about twice as large as those based on *F*, and *R*- factors based on ALL data will be even larger.

### Fractional atomic coordinates and isotropic or equivalent isotropic displacement parameters (Å^2^)

**Table:**

	*x*	*y*	*z*	*U*_iso_*/*U*_eq_	
O1	0.5955 (4)	0.7958 (3)	0.22054 (17)	0.0794 (8)	
O2	0.7633 (4)	0.9522 (3)	0.20430 (17)	0.0834 (9)	
O3	−0.0370 (4)	1.0058 (2)	0.39049 (17)	0.0710 (8)	
H31	−0.124 (8)	0.969 (4)	0.410 (3)	0.118 (18)*	
O4	0.0269 (3)	0.8270 (2)	0.34367 (15)	0.0641 (7)	
O5	0.6973 (3)	0.9189 (2)	0.45814 (16)	0.0722 (8)	
O6	0.3065 (6)	0.4261 (2)	0.4071 (2)	0.1102 (12)	
O7	0.0643 (5)	0.5349 (3)	0.4190 (2)	0.1020 (11)	
N1	0.6283 (4)	0.9028 (3)	0.22704 (15)	0.0593 (8)	
N2	0.5917 (4)	0.8218 (2)	0.45025 (15)	0.0507 (7)	
N3	0.2371 (6)	0.5211 (3)	0.42153 (18)	0.0710 (9)	
C1	0.5010 (4)	0.9786 (3)	0.26571 (16)	0.0459 (7)	
C2	0.5435 (5)	1.0985 (3)	0.27801 (19)	0.0561 (8)	
H2	0.6492	1.1328	0.2614	0.067*	
C3	0.4256 (5)	1.1666 (3)	0.3156 (2)	0.0601 (9)	
H3	0.4515	1.2482	0.3244	0.072*	
C4	0.2683 (5)	1.1149 (3)	0.34055 (19)	0.0536 (8)	
H4	0.1901	1.1615	0.3663	0.064*	
C5	0.2281 (4)	0.9940 (3)	0.32703 (16)	0.0439 (7)	
C6	0.3461 (4)	0.9239 (3)	0.28935 (17)	0.0462 (7)	
H6	0.3213	0.8423	0.2803	0.055*	
C7	0.0623 (4)	0.9326 (3)	0.35400 (17)	0.0484 (7)	
C8	0.3601 (5)	0.6276 (3)	0.43511 (18)	0.0511 (8)	
C9	0.5191 (5)	0.6308 (3)	0.3996 (2)	0.0616 (9)	
H9	0.5471	0.5661	0.3700	0.074*	
C10	0.6347 (5)	0.7289 (3)	0.40788 (19)	0.0589 (9)	
H10	0.7427	0.7316	0.3844	0.071*	
C11	0.4381 (4)	0.8176 (3)	0.48658 (17)	0.0487 (8)	
H11	0.4143	0.8828	0.5165	0.058*	
C12	0.3155 (4)	0.7205 (3)	0.48104 (17)	0.0474 (7)	
C13	0.1538 (5)	0.7242 (3)	0.5254 (2)	0.0644 (9)	
H131	0.1737	0.7882	0.5617	0.077*	
H132	0.1457	0.6485	0.5508	0.077*	
H133	0.0379	0.7383	0.4922	0.077*	

### Atomic displacement parameters (Å^2^)

**Table:**

	*U*^11^	*U*^22^	*U*^33^	*U*^12^	*U*^13^	*U*^23^
O1	0.0657 (17)	0.0703 (17)	0.110 (2)	0.0069 (14)	0.0378 (15)	−0.0094 (15)
O2	0.0574 (15)	0.110 (2)	0.091 (2)	−0.0030 (14)	0.0417 (14)	0.0116 (15)
O3	0.0590 (15)	0.0623 (14)	0.102 (2)	−0.0044 (13)	0.0461 (15)	−0.0127 (13)
O4	0.0551 (14)	0.0555 (14)	0.0876 (18)	−0.0110 (11)	0.0306 (13)	−0.0063 (11)
O5	0.0588 (15)	0.0717 (15)	0.0941 (19)	−0.0270 (13)	0.0386 (14)	−0.0219 (13)
O6	0.145 (3)	0.0527 (16)	0.133 (3)	−0.0154 (19)	0.021 (2)	−0.0251 (17)
O7	0.087 (2)	0.101 (2)	0.122 (3)	−0.0437 (19)	0.028 (2)	−0.0185 (18)
N1	0.0429 (15)	0.077 (2)	0.0615 (18)	0.0043 (14)	0.0185 (13)	0.0065 (14)
N2	0.0415 (14)	0.0544 (15)	0.0592 (16)	−0.0065 (12)	0.0173 (12)	−0.0074 (11)
N3	0.093 (3)	0.0560 (18)	0.0660 (18)	−0.0205 (18)	0.0176 (18)	−0.0085 (14)
C1	0.0365 (15)	0.0542 (17)	0.0485 (16)	0.0003 (13)	0.0110 (13)	0.0057 (13)
C2	0.0469 (18)	0.0591 (19)	0.063 (2)	−0.0071 (15)	0.0113 (16)	0.0153 (15)
C3	0.061 (2)	0.0433 (16)	0.077 (2)	−0.0100 (15)	0.0142 (18)	0.0037 (15)
C4	0.0505 (18)	0.0485 (17)	0.064 (2)	0.0046 (15)	0.0150 (16)	0.0028 (14)
C5	0.0361 (15)	0.0479 (16)	0.0483 (16)	0.0003 (13)	0.0081 (13)	0.0057 (12)
C6	0.0394 (15)	0.0466 (16)	0.0541 (17)	−0.0022 (13)	0.0115 (13)	0.0021 (12)
C7	0.0386 (16)	0.0529 (18)	0.0550 (17)	−0.0047 (14)	0.0116 (14)	−0.0038 (14)
C8	0.0540 (18)	0.0473 (16)	0.0536 (18)	−0.0070 (15)	0.0127 (15)	−0.0036 (13)
C9	0.072 (2)	0.0547 (19)	0.063 (2)	0.0009 (17)	0.0244 (18)	−0.0128 (15)
C10	0.0532 (19)	0.065 (2)	0.064 (2)	0.0027 (17)	0.0264 (16)	−0.0085 (16)
C11	0.0430 (16)	0.0518 (17)	0.0542 (18)	−0.0050 (14)	0.0175 (14)	−0.0094 (13)
C12	0.0430 (16)	0.0498 (16)	0.0512 (17)	−0.0048 (14)	0.0128 (13)	−0.0001 (13)
C13	0.055 (2)	0.069 (2)	0.074 (2)	−0.0162 (17)	0.0272 (18)	−0.0095 (17)

### Geometric parameters (Å, °)

**Table:**

O1—N1	1.209 (4)	C3—H3	0.9300
O2—N1	1.226 (3)	C4—C5	1.383 (4)
O3—C7	1.311 (4)	C4—H4	0.9300
O3—H31	0.86 (5)	C5—C6	1.389 (4)
O4—C7	1.205 (3)	C5—C7	1.502 (4)
O5—N2	1.307 (3)	C6—H6	0.9300
O6—N3	1.206 (4)	C8—C9	1.378 (5)
O7—N3	1.234 (4)	C8—C12	1.384 (4)
N1—C1	1.480 (4)	C9—C10	1.358 (5)
N2—C10	1.341 (4)	C9—H9	0.9300
N2—C11	1.352 (4)	C10—H10	0.9300
N3—C8	1.468 (4)	C11—C12	1.380 (4)
C1—C2	1.372 (4)	C11—H11	0.9300
C1—C6	1.377 (4)	C12—C13	1.492 (4)
C2—C3	1.376 (5)	C13—H131	0.9600
C2—H2	0.9300	C13—H132	0.9600
C3—C4	1.388 (5)	C13—H133	0.9600
			
C7—O3—H31	113 (3)	C1—C6—H6	120.9
O1—N1—O2	123.7 (3)	C5—C6—H6	120.9
O1—N1—C1	118.5 (3)	O4—C7—O3	124.1 (3)
O2—N1—C1	117.8 (3)	O4—C7—C5	123.1 (3)
O5—N2—C10	121.2 (3)	O3—C7—C5	112.8 (3)
O5—N2—C11	117.9 (2)	C9—C8—C12	122.1 (3)
C10—N2—C11	120.9 (3)	C9—C8—N3	117.0 (3)
O6—N3—O7	122.5 (4)	C12—C8—N3	120.9 (3)
O6—N3—C8	118.8 (4)	C10—C9—C8	119.7 (3)
O7—N3—C8	118.4 (3)	C10—C9—H9	120.2
C2—C1—C6	122.9 (3)	C8—C9—H9	120.2
C2—C1—N1	119.2 (3)	N2—C10—C9	119.4 (3)
C6—C1—N1	117.8 (3)	N2—C10—H10	120.3
C1—C2—C3	118.2 (3)	C9—C10—H10	120.3
C1—C2—H2	120.9	N2—C11—C12	122.6 (3)
C3—C2—H2	120.9	N2—C11—H11	118.7
C2—C3—C4	120.7 (3)	C12—C11—H11	118.7
C2—C3—H3	119.7	C11—C12—C8	115.2 (3)
C4—C3—H3	119.7	C11—C12—C13	117.9 (3)
C5—C4—C3	119.9 (3)	C8—C12—C13	126.9 (3)
C5—C4—H4	120.1	C12—C13—H131	109.5
C3—C4—H4	120.1	C12—C13—H132	109.5
C4—C5—C6	120.1 (3)	H131—C13—H132	109.5
C4—C5—C7	122.2 (3)	C12—C13—H133	109.5
C6—C5—C7	117.6 (3)	H131—C13—H133	109.5
C1—C6—C5	118.2 (3)	H132—C13—H133	109.5
			
O1—N1—C1—C2	175.7 (3)	O6—N3—C8—C9	28.6 (5)
O2—N1—C1—C2	−3.3 (4)	O7—N3—C8—C9	−145.4 (4)
O1—N1—C1—C6	−2.9 (4)	O6—N3—C8—C12	−152.1 (4)
O2—N1—C1—C6	178.1 (3)	O7—N3—C8—C12	34.0 (5)
C6—C1—C2—C3	−0.1 (5)	C12—C8—C9—C10	−1.6 (5)
N1—C1—C2—C3	−178.5 (3)	N3—C8—C9—C10	177.7 (3)
C1—C2—C3—C4	0.2 (5)	O5—N2—C10—C9	−178.4 (3)
C2—C3—C4—C5	−0.6 (5)	C11—N2—C10—C9	2.0 (5)
C3—C4—C5—C6	0.8 (5)	C8—C9—C10—N2	−0.4 (5)
C3—C4—C5—C7	178.8 (3)	O5—N2—C11—C12	178.8 (3)
C2—C1—C6—C5	0.2 (5)	C10—N2—C11—C12	−1.6 (5)
N1—C1—C6—C5	178.7 (3)	N2—C11—C12—C8	−0.4 (4)
C4—C5—C6—C1	−0.6 (4)	N2—C11—C12—C13	178.3 (3)
C7—C5—C6—C1	−178.7 (3)	C9—C8—C12—C11	2.0 (5)
C4—C5—C7—O4	−178.9 (3)	N3—C8—C12—C11	−177.3 (3)
C6—C5—C7—O4	−0.9 (5)	C9—C8—C12—C13	−176.6 (3)
C4—C5—C7—O3	0.3 (4)	N3—C8—C12—C13	4.1 (5)
C6—C5—C7—O3	178.3 (3)		

### Hydrogen-bond geometry (Å, °)

**Table:**

*D*—H···*A*	*D*—H	H···*A*	*D*···*A*	*D*—H···*A*
O3—H31···O5^i^	0.86 (5)	1.73 (6)	2.578 (3)	170 (5)
C10—H10···O4^ii^	0.93	2.48	3.351 (4)	156
C2—H2···O1^iii^	0.93	2.55	3.371 (4)	148
C9—H9···O2^iv^	0.93	2.39	3.260 (4)	156
C13—H131···O3^v^	0.96	2.67	3.502 (4)	146
C11—H11···O5^vi^	0.93	2.40	3.270 (4)	156
C13—H132···O7^vii^	0.96	2.62	3.475 (4)	148

Symmetry codes: (i) *x*−1, *y*, *z*; (ii) *x*+1, *y*, *z*; (iii) −*x*+3/2, *y*+1/2, −*z*+1/2; (iv) −*x*+3/2, *y*−1/2, −*z*+1/2; (v) −*x*, −*y*+2, −*z*+1; (vi) −*x*+1, −*y*+2, −*z*+1; (vii) −*x*, −*y*+1, −*z*+1.
